# Ozone-induced stomatal sluggishness changes carbon and water balance of temperate deciduous forests

**DOI:** 10.1038/srep09871

**Published:** 2015-05-06

**Authors:** Yasutomo Hoshika, Genki Katata, Makoto Deushi, Makoto Watanabe, Takayoshi Koike, Elena Paoletti

**Affiliations:** 1 Silviculture and Forest Ecological Studies, Hokkaido University, Sapporo 060-8689, Japan; 2 (present address) Institute of Sustainable Plant Protection, National Research Council of Italy, Via Madonna del Piano, I-50019 Sesto Fiorentino, Florence, Italy; 3 Research Group for Environmental Science, Japan Atomic Energy Agency*, 2-4 Shirakata-Shirane, Tokai, Naka, Ibaraki*, 319-1195 Japan; 4 (present address) Atmospheric Environmental Research, Institute of Meteorology and Climate Research, Karlsruhe Institute of Technology, Kreuzeckbahnstr. 19, 82467, Garmisch-Partenkirchen, Germany; 5 Institute of Agriculture, Tokyo University of Agriculture and Technology, Fuchu 183-8509, Japan; 6 Atmospheric Environment and Applied Meteorology Research Department, Meteorological Research Institute, Tsukuba, Japan

## Abstract

Tropospheric ozone concentrations have increased by 60–100% in the Northern Hemisphere since the 19^th^ century. The phytotoxic nature of ozone can impair forest productivity. In addition, ozone affects stomatal functions, by both favoring stomatal closure and impairing stomatal control. Ozone-induced stomatal sluggishness, i.e., a delay in stomatal responses to fluctuating stimuli, has the potential to change the carbon and water balance of forests. This effect has to be included in models for ozone risk assessment. Here we examine the effects of ozone-induced stomatal sluggishness on carbon assimilation and transpiration of temperate deciduous forests in the Northern Hemisphere in 2006-2009 by combining a detailed multi-layer land surface model and a global atmospheric chemistry model. An analysis of results by ozone FACE (Free-Air Controlled Exposure) experiments suggested that ozone-induced stomatal sluggishness can be incorporated into modelling based on a simple parameter (*g*_min_, minimum stomatal conductance) which is used in the coupled photosynthesis-stomatal model. Our simulation showed that ozone can decrease water use efficiency, i.e., the ratio of net CO_2_ assimilation to transpiration, of temperate deciduous forests up to 20% when ozone-induced stomatal sluggishness is considered, and up to only 5% when the stomatal sluggishness is neglected.

Tropospheric ozone (O_3_) is recognized as a significant phytotoxic air pollutant and greenhouse gas^1^, formed from photochemical reactions of its precursors such as nitrogen oxides and volatile organic compounds^2^. Ozone concentrations have increased by approximately 60–100% since pre-industrial times in the Northern Hemisphere^3^,^4^,^5^. Ozone is considered to be one of the most important factors affecting forest health^6^.

Stomata, i.e., small pores on leaves, are a crucial interface for gas exchange between forests and the atmosphere. Ozone enters plants via stomata and causes a decline of photosynthetic capacity^6^. In addition, O_3_ is generally known to induce stomatal closure^7^, which results in reduced O_3_ uptake by plants and water saving due to less transpiration. In parallel, O_3_ exposure also causes slow or less efficient stomatal control (O_3_-induced stomatal sluggishness)^7^, which results in incomplete stomatal closure e.g. under low light conditions (i.e., leaky stomata). This may lead to further O_3_ uptake and water consumption. Ozone-induced stomatal sluggishness has been reported in many temperate tree species^7^,^8^,^9^,^10^,^11^. Existing models for O_3_ risk assessment in forests have included O_3_ effect on stomata as a decrease in stomatal conductance proportional to the O_3_-induced decline of photosynthesis^5^,^12^, while the effect of O_3_-induced stomatal sluggishness has been usually neglected. This sluggish response along with O_3_-impaired photosynthesis may significantly change the water and carbon balance of forests under a changing environment^4^. Lombardozzi *et al*.^9^ included O_3_-induced stomatal sluggishness in a global biosphere model where the data were from chamber experiments on tulip poplar and a constant 100 nmol mol^–^^1^ O_3_ concentration across the world was simulated. They suggested that O_3_-induced stomatal sluggishness may ameliorate the O_3_-induced decline of carbon assimilation and transpiration of trees^9^. However, the environmental conditions in the chambers (e.g., enhanced air temperature, high air ventilation) are known to change plant responses to O_3_ relative to actual field conditions^13^. Therefore, the results have to be verified based on more realistic data from technologies such as the recently developed O_3_-FACE (Free-Air Controlled Exposure) approach^14^. In this study, we used O_3_-FACE data, and estimated O_3_-induced stomatal sluggishness implications for carbon assimilation and transpiration. We focused on O_3_-sensitive temperate deciduous forests exposed to realistic O_3_ concentrations in the Northern Hemisphere.

As postulated by two recent studies^15^,^16^, we considered the following new concept for modelling O_3_ effects on stomata: 1) stomata close in tandem with the O_3_-induced decline of photosynthesis, and 2) stomatal response to environmental variables is impaired due to O_3_-induced stomatal sluggishness. We then investigated how O_3_ uptake changed the parameters of the photosynthesis-stomatal model (the Ball-Woodrow-Berry model^17^, see *Methods*), which is widely used in many land-surface schemes in climate models^5^,^9^. Reliable O_3_-FACE datasets for modelling O_3_-induced stomatal sluggishness along the forest growing season are currently very limited. To our knowledge, a reliable dataset for analyzing the relationship between O_3_-induced stomatal sluggishness and O_3_ uptake over the growing season is available from our previous work^18^, which investigated the seasonal change of stomatal conductance of Siebold’s beech (*Fagus crenata*) under free-air O_3_ exposure (O_3_-FACE) in Japan. Using this dataset, we derived the parameters of the Ball-Woodrow-Berry model for assessing O_3_-induced stomatal sluggishness. To verify our result, we analyzed literature values of stomatal conductance from another O_3_-FACE experiment with trees, i.e., the Aspen FACE^19^, and estimated the Ball-Woodrow-Berry model parameters. Although we could not analyze the relationship between O_3_-induced stomatal sluggishness and O_3_ uptake using the Aspen FACE data due to the limitation of the measurement period (only once in July), an intercomparison of the results allowed us to validate the parameters of O_3_-induced stomatal sluggishness. Finally, using the parameters for Siebold’s beech, the impact of O_3_-induced stomatal sluggishness on net CO_2_ assimilation and transpiration in temperate deciduous forests in the Northern Hemisphere was calculated by offline (one-way) coupling simulations of a multi-layer atmosphere-SOil-VEGetation model (SOLVEG)^20^,^21^ and Meteorological Research Institute Chemistry-Climate Model version 2 (MRI-CCM2)^22^,^23^. Three SOLVEG simulations were carried out: 1) including no O_3_ effect (“control run”), 2) including O_3_ effects on photosynthesis without O_3_-induced stomatal sluggishness (“no sluggishness run”) and 3) including O_3_ effects on photosynthesis with O_3_-induced stomatal sluggishness (“sluggishness run”) (see *Methods*). Ozone-induced changes of net CO_2_ assimilation, transpiration, and water use efficiency (WUE), i.e., the ratio of net CO_2_ assimilation to transpiration, were assessed by the ratio of differences between “sluggishness run” or “no sluggishness run” and “control run”.

## Results and Discussion

**Figure 1:**
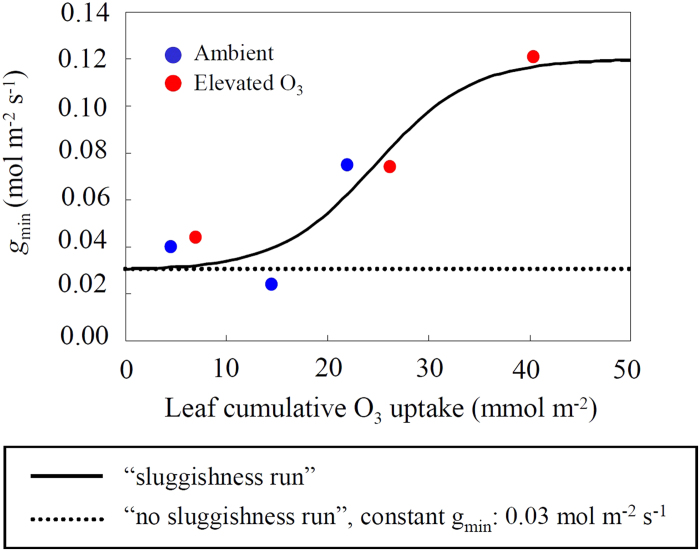
**Changes of *g*_min_ over a range of cumulative O_3_ uptake (*CUO*) used for the “sluggishness run” and “no sluggishness run” of SOLVEG-MRI-CCM2.** Data points of *g*_min_ were obtained from an analysis of measurements in June, August and October 2012 (see Fig. S1) at the O_3_-FACE experiment on Siebold’s beech in Japan (blue circle: ambient O_3_; red circle: elevated O_3_). Obtained *g*_min_ were fitted by a sigmoid function for “sluggishness run” (solid line): *g*_min_ = 0.03 + 0.09/[1 + *exp*{–0.21·(CUO – 24.7)}], *R*^2^ = 0.89. Dashed line shows no change of *g*_min_ (*g*_min_ = 0.03 mol m^−2^ s^−1^) and was used for “no sluggishness run”.

Our study suggests a simple way to include O_3_-induced stomatal sluggishness in the Ball-Woodrow-Berry model. This model has two empirical parameters (see Eq. 1 in *Methods*): *m*, slope of the linear relationship between stomatal conductance and photosynthesis; and *g*_min_, y-intercept of this relationship. We found that *g*_min_ of Siebold’s beech increased due to an increase of cumulative O_3_ uptake (Fig. 1), while there was no significant relationship between *m* and cumulative O_3_ uptake (data not shown, linear regression analysis, *p* = 0.329). This enhanced *g*_min_ after O_3_ exposure (Fig. 1, S1B, S1C) was supported by the analysis of literature data from Aspen FACE (*g*_min_ was 0.034 mol m^−2^ s^−1^ in ambient air and 0.100 mol m^−2^ s^−1^ at elevated O_3_, Fig. S2). Ozone is generally known to cause a reduction of WUE^5^. An increase of *g*_min_ without change of *m* indicates a reduction of WUE at elevated O_3_ compared to ambient conditions (Fig. S1B, S1C, S2). The enhanced *g*_min_ can be considered as slowed stomatal closure to decreasing light intensity under elevated O_3_^7^,^11^. This implies imperfect stomatal closing under low light conditions^18^ and impaired control on water loss^7^.

**Figure 2:**
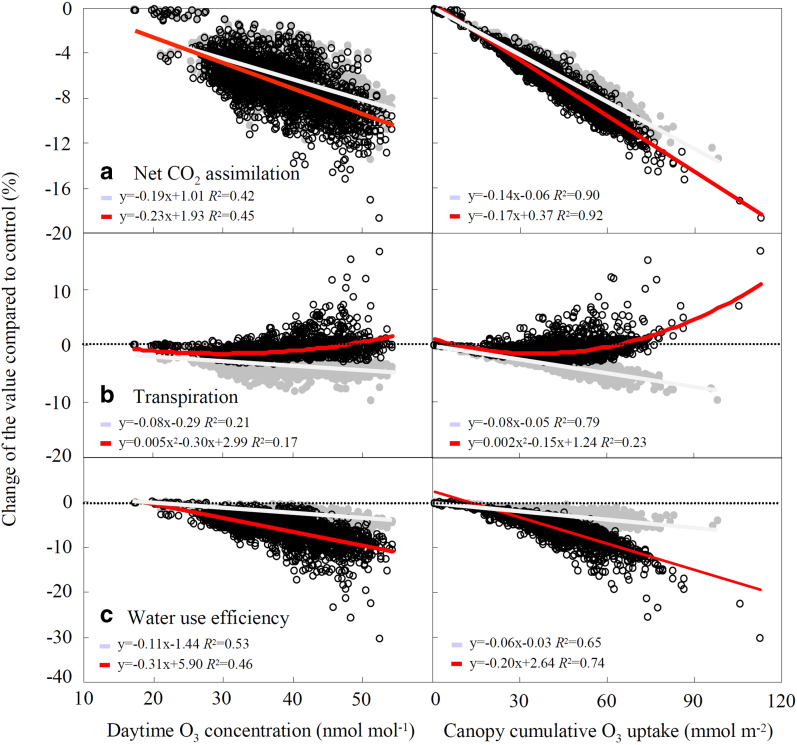
**Percent change of modelled net CO_2_ assimilation, transpiration and water use efficiency in temperate deciduous forests in the Northern Hemisphere in relation to daytime mean O_3_ concentration or cumulative canopy O_3_ uptake (years 2006-2009).** a, net CO_2_ assimilation, b, transpiration, and c, water use efficiency were simulated by the offline coupling simulation of SOLVEG-MRI-CCM2. Effects of O_3_-induced stomatal sluggishness were included (black open circles and red lines) or excluded (gray circles and gray lines). The percentage of change of each parameter was calculated relative to “control run” (no O_3_ effect).

The novel parameterization of *g*_min_ shown in Fig. 1 was then applied to simulate the O_3_ effect on carbon and water balances in temperate deciduous forests in the Northern Hemisphere. Those forests are dominated by oak, poplar and beech species^24^. While oaks are usually O_3_ tolerant species^12^, we investigated the response of two species, beech and aspen, that are O_3_-sensitive^5^,^12^ and representative of late and early successional forests, respectively. So our simulations explored the impact of O_3_ on carbon and water balance in O_3_–sensitive temperate deciduous forests of the Northern Hemisphere. The offline coupling simulations of SOLVEG and MRI-CCM2 revealed that net CO_2_ assimilation declined with an increase of O_3_ exposure (Fig. 2a) and of canopy cumulative O_3_ uptake (Fig. 2b). The O_3_-induced decline of net CO_2_ assimilation at the average daytime O_3_ concentrations of 37.2 ± 6.2 nmol mol^−1^ was 6.6 ± 2.1% and 6.0 ± 1.8% in the “sluggishness run” and “no sluggishness run”, respectively (Figs. 2a, 2b) as an average of all years and grids where temperate deciduous forests occurred (Fig. S3). Therefore, O_3_-induced stomatal sluggishness did not ameliorate the effect of O_3_ on carbon assimilation of trees as suggested by Lombardozzi *et al*.^9^. Higher O_3_ concentrations, e.g., 44.6 ± 4.7 nmol mol^−1^ as an average of daytime values over China, resulted in 9.1 ± 2.0% and 8.0 ± 1.6% reductions in the “sluggishness run” and “no sluggishness run”, respectively. Such a stronger impact on carbon assimilation, when O_3_-induced stomatal sluggishness was included, was due to enhanced stomatal O_3_ uptake, which led to a further negative impact on photosynthesis.

The “no sluggishness run” predicted a monotonic reduction of transpiration by stomatal closure under elevated O_3_, in tandem with declining carbon assimilation (Fig. 2b, gray line), as reported in state-of-art global climate models^5^. In contrast, the “sluggishness run” showed a decrease of transpiration until 30 nmol mol^−1^ of O_3_ concentration or 37 mmol m^−2^ of canopy cumulative O_3_ uptake, and then an increase with increasing O_3_ exposure or uptake (Fig. 2b, red line). This suggests that the tight coupling of stomatal conductance and photosynthesis at low O_3_ environment cannot be maintained at higher O_3_ pollution, and results in increasing transpirational water loss due to sluggish stomata. As a result, O_3_–induced reduction of transpiration at the average daytime O_3_ concentration of 37.2 ± 6.2 nmol mol^−1^ was only 1.0 ± 1.4% in the “sluggishness run”, while a larger decline (3.4 ± 1.1%) was found in the “no sluggishness run” (Fig. 2b). At higher O_3_ concentrations, e.g., 44.6 ± 4.7 nmol mol^−1^ as an average of daytime values over China, the decline was 0.3 ± 1.6% and 4.3 ± 1.0% in the “sluggishness run” and “no sluggishness run”, respectively. In agreement with a meta-analytic review by Lombardozzi *et al*.^25^, our “sluggishness run” thus suggests that O_3_ reduces carbon assimilation more than transpiration (Figs. 2a, 2b). The “sluggishness run” can also explain the increase of transpiration measured by sap-flow at the Aspen FACE experiment (~18% in late summer under elevated O_3_ relative to control)^15^ and can justify the reduced late-season streamflow of forest watersheds under regionally elevated O_3_ exposure on the Appalachian foothills of the USA^15^.

Ozone decreased WUE in both the “sluggishness run” and “no sluggishness run” (Fig. 2c). A larger decline of WUE per unit O_3_ exposure or uptake, however, was found in the “sluggishness run” (up to 20%) relative to “no sluggishness run” (up to 5%) (Fig. 2c). Our result suggests that O_3_-induced stomatal sluggishness can significantly change forest carbon and water balances. This change partly explains the trend of forest WUE as observed at flux sites in North America^26^. Keenan *et al*.^26^ recently reported that forest WUE in North America increased over the last 15 years (approximately + 30%), and concluded that this increase resulted from increasing ambient CO_2_ concentration. The increase of WUE, however, was much greater than expected from theoretical and experimental evidence regarding plant response to CO_2_^27^. Holmes^28^ pointed out that a decrease of daytime mean O_3_ concentration at North American forest sites (8–10 nmol mol^−1^ during the last 15 years), may partly explain the WUE trend (maximum 3-4% increase of WUE), based on literature data of WUE response to O_3_. According to our “sluggishness run”(Fig. 2c), we estimated a 2-3% increase of WUE by a 8–10 nmol mol^−1^ decrease in O_3_ concentrations, while only a ~1% increase of WUE was found in the “no sluggishness run” (Fig. 2c). This result suggests that a significant part of the WUE trend at North American sites (corresponding to about one-tenth of the observed WUE trend) may be explained by O_3_ effects, when O_3_-induced stomatal sluggishness is included.

**Figure 3:**
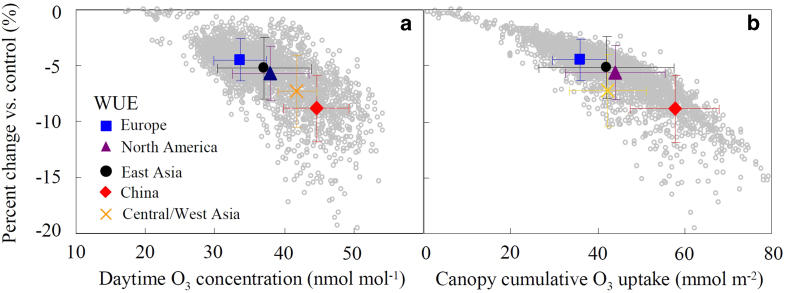
**Percent changes of reduction in modelled water use efficiency (WUE) in relation to daytime mean O_3_ concentration or canopy cumulative O_3_ uptake for several regions in the Northern Hemisphere (years 2006–2009).** The percentage of reduction of WUE relative to “control run” (no O_3_ effect) was calculated by the offline coupling simulation of SOLVEG-MRI-CCM2 including O_3_-induced stomatal sluggishness. Plots and bars represent mean values and standard deviations, respectively. The five regions, i.e., Europe, North America, East Asia (without China), China and Central/West Asia are defined in Fig. S3.

According to our “sluggishness run”, the contribution of O_3_ to the decline in WUE ranged from 4.5 ± 1.9 to 8.8 ± 3.0% in different regions of the Northern Hemisphere (Fig. 3, S8). For example, the maximum difference (−8.8%) was found in China (Fig. 3), where relatively high level of O_3_ concentrations were shown (45 nmol mol^−1^ as daytime average, Fig. S4). Water limiting conditions (approximately 350 mm of precipitation from 1 May to 1 November from 2006–2009, data not shown) may decrease canopy cumulative O_3_ uptake in Central/West Asia (Fig. 3b) and reduce O_3_-induced impairment of forest WUE, even though O_3_ concentrations were relatively high (42 nmol mol^−1^ as daytime average) in this region (Fig. 3a).

In contrast with the frequent assumption that O_3_ reduces tree water use^29^, we demonstrated that O_3_-induced stomatal sluggishness has a potential to increase transpiration and thus explain observational evidence of reduced streamflow from forests^15^. Less efficient water use is expected to increase susceptibility of forest trees to drought and fire, which in turn are expected to increase in frequency and intensity due to climate change^1^. In addition to reduced carbon sequestration^5^,^29^, O_3_-increased transpiration can elevate air humidity and radiative forcing of water vapour relative to current estimates by global climate models^9^. Although further works are needed to assess the real-world impacts (e.g., verification of modelled O_3_ concentration, and pre-industrial and future simulations), our results revealed that O_3_-induced stomatal sluggishness cannot be ignored when modelling biosphere-atmosphere interactions. This implies that stomatal sluggishness is essential to assessing impacts of air quality to terrestrial ecosystems under present and future atmospheric conditions.

## Methods

### A parameterization of stomatal conductance

Our parameterization of O_3_-induced stomatal sluggishness was based on leaf gas exchange data^18^ obtained from an O_3_-FACE experiment in Sapporo Experimental Forest, Hokkaido University, in northern Japan (43°04' N, 141°20' E, 15 m a.s.l., annual mean temperature: 9.3˚C, total precipitation: 1279 mm in 2012). Details of the exposure system are available in a previous paper^30^. Ozone was generated from pure oxygen by an O_3_ generator (Model PZ-1C, Kofloc, Kyoto, Japan). The resulting O_3_ was diluted with ambient air in a mixing tank and passed into the canopies through fluorine resin tubes hanging from a fixed grid above the trees down to a height of 50 cm above the ground. The target O_3_ concentration above the canopy was 60 nmol mol^−1^ during daylight hours. This concentrations of O_3_ corresponded to the legislative standard for O_3_ in Japan, where similar or higher O_3_ concentrations have often been observed in many regions, including forested areas^31^. This target concentration was also consistent with the level of elevated O_3_ concentrations applied in previous O_3_-FACE experiments^14^. This enhanced daytime O_3_ treatment was applied from August to November 2011, and from May to November 2012. The daytime hourly mean O_3_ concentrations in ambient and elevated O_3_ were 25.7 ± 11.4 nmol mol^−1^ and 56.7 ± 10.5 nmol mol^−1^ in 2011, and 27.5 ± 11.6 nmol mol^−1^ and 61.5 ± 13.0 nmol mol^−1^ in 2012. The average volumetric soil water content was large enough to avoid the water stress to trees (28.1 ± 2.8%).

We focused on Siebold’s beech, which is an O_3_ sensitive tree species^30^ widely distributed in cool-temperate climate. Diurnal course of leaf gas exchange was measured in fully expanded leaves exposed to full sun at the top of the canopy, using a portable infra-red gas analyzer (Model 6400, Li-Cor instruments, Lincoln, NE, USA) in June, August and October 2012 (see ref.^18^ for a detail). No difference in leaf gas exchange of Siebold’s beech between ambient and elevated O_3_ treatments was found before the start of fumigation^11^. There was also no difference in the leaf nitrogen content in August 2012^18^. Measured data in each month were used to estimate the parameters of the Ball-Woodrow-Berry stomatal conductance model^17^ as follows:


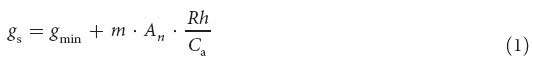


where *g*_min_ is the minimum stomatal conductance (mol m^−2^ s^−1^), *m* is the Ball-Woodrow-Berry slope of the conductance-photosynthesis relationship (no dimension), *A*_*n*_ is net photosynthetic rate (μmol m^−2^ s^−1^), *Rh* is relative humidity at the leaf surface (no dimension), and *C*_a_ is CO_2_ concentration at the leaf surface (μmol mol^−1^). We parameterized monthly *g*_min_ as a function of cumulative O_3_ uptake of a leaf (*CUO*, mmol m^−2^) (Fig. 1), which was estimated by the DO_3_SE model (the fully empirical multiplicative stomatal conductance model) using meteorological and O_3_ concentration data at the experimental site. In contrast with the Ball-Woodrow-Berry model, the DO_3_SE model does not consider photosynthesis, and modifies a reference value of stomatal conductance (denoted as maximum stomatal conductance, *g*_max_) according to changes of environmental variables (i.e., light intensity, temperature, atmospheric humidity, and soil moisture). In our previous study, the DO_3_SE model was parameterized by using data recorded for Siebold’s beech and by including O_3_ effects on stomatal conductance. The model showed a good agreement with the observation of stomatal conductance (*R*^2^ = 0.68).

To verify our result, we analyzed literature stomatal conductance data at Aspen FACE^19^, where terminal and lateral shoots from the upper and lower crown of an O_3_ sensitive aspen clone were measured in July after two years of O_3_ exposure to 55 nmol mol^−1^. Data points were obtained using the image analysis software SimpleDigitizer 3.2 (Haruyuki Fujimaki, Tokyo, Japan). Relative humidity (*Rh*) and CO2 concentration at the leaf surface (*C*_*a*_) were set to 50% and 360 μmol mol^−1^, respectively, according to Noormets *et al*.^19^ We then calculated *m* and *g*_min_ at Aspen FACE (Fig. S2), and compared the results with those at the O_3_-FACE in Japan.

### Modelling ozone effect in SOLVEG

We modified the parameters for calculating net photosynthetic rate (*A*_*n*_) and stomatal conductance (*g*_*s*_) in SOLVEG in order to account for O_3_ effects based on *CUO*. SOLVEG calculates the O_3_ deposition flux (*F*_O3_) at each canopy layer (Supplementary Eq. (S1)) using stomatal resistance (*r*_*s*_) which equals the reciprocal of *g*_*s*_, and quasi-laminar resistance over the leaves (*r*_*b*_)^20^,^21^:


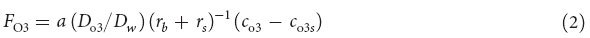


where *a* (m^2^ m^−3^) is the leaf area density (LAD) at the canopy layer, *D*_*o3*_ and *D*_*w*_ (m^2^ s^−1^) are the diffusivities of O_3_ and water vapor, respectively, and *c*_O3_ and *c*_O3*s*_ (nmol mol^−1^) are the O_3_ concentrations in the canopy layer and sub-stomatal cavity, respectively. For simplicity, it was assumed that *c*_O3*s*_ = 0 in Eq. (2) (ref. 32). *CUO* was calculated as temporal accumulation of *F*_*O3*_ at each canopy layer.

To calculate *r*_*s*_ in Eq. (2), SOLVEG requires the maximum catalytic capacity of the photosynthetic enzyme system Ribulose-1,5-bisphosphate carboxylase/oxygenase (Rubisco), *V*_*cmax*_, at 25°C [*V*_*cmax25*_; Supplementary Eqs. (S2)–(S5)], *m*, and *g*_*min*_ as input parameters. In this study, all parameters were obtained from the results for Siebold’s beech in the O_3_ FACE in Japan^18^. The following fitting curves against *CUO* were applied to *V*_*cmax25*_ and *g*_*min*_: *V*_*cmax25*_ = –0.887·*CUO* + 62.95 (Eq. 3; *R*^2^ = 0.94, *p* = 0.001), *g*_*min*_ = 0.03 + 0.09/[1 + *exp*{–0.21·(CUO – 24.7)}] (Eq. 4; Fig. 1, solid lines). The value of *m* was set to 15, which was the mean value during the experimental period. Ozone uptake at each canopy layer calculated by Eq. (2) was vertically integrated for all canopy layers to obtain canopy-scale O_3_ flux.

### Simulation conditions of SOLVEG and MRI-CCM2

To investigate the O_3_ effects under different climate conditions, SOLVEG was applied to each horizontal grid of MRI-CCM2 temperate deciduous forests in the Northern Hemisphere (Fig. S3). The Japanese 55-year Reanalysis (JRA55: in the horizontal, 1.25° latitude/longitude regular grid resolution (The original horizontal resolution of JRA55 is a spectral triangular 319 with a reduced Gaussian grid, roughly equivalent to 0.5625° × 0.5625° lat-lon); in the vertical, 60 layers (L60) from the surface to 0.1 hPa)^33^ was used for input data to force the initial and upper boundary conditions of meteorological variables (atmospheric pressure, downward short- and long-wave radiations, precipitation, wind speed, and air temperature and humidity near the surface) in SOLVEG. The parameters of canopy structures (leaf area index and canopy height) and CO_2_ concentration were set to be typical values (Table S1). Soil type was set to typical loam for all horizontal grids based on the harmonized world soil database^34^. At the initial day of calculations at each calculation year (1 May), soil temperature and moisture were given to each depth of soil by vertically interpolating the values of JRA55 data at the surface and bottom of the soil. Three sets of SOLVEG runs were carried out: 1) including both reduction of *V*_*cmax*25_ (i.e., O_3_-induced decline of photosynthesis) and increase of *g*_*min*_ due to O_3_-induced stomatal sluggishness with an increase of *CUO* (“sluggishness run”), 2) including the reduction of *V*_*cmax*25_ only (“no sluggishness run”), and 3) including no ozone effect (“control run”). The percentage variations of net CO_2_ assimilation, transpiration, and WUE were calculated as the ratio of differences between “sluggishness run” or “no sluggishness run” and “control run”.

The MRI-CCM2 is used to generate 3-hourly averaged surface ozone concentration for SOLVEG calculations. The MRI-CCM2^22^,^23^ is a global chemistry-climate model, in which an atmospheric chemistry model is coupled to the MRI’s latest atmospheric general circulation model (MRI-AGCM3) via a simple coupler. MRI-CCM2 was run for the period 2005–2009, and the simulation results between 2006 and 2009 were used for the SOLVEG calculations. In the simulation, the horizontal wind field was nudged toward JRA55 by using a Newtonian relaxation technique with a 24 h e-folding time. The horizontal spectral resolution was set to TL159, corresponding to a grid size of about 120 km. In the vertical, the model had 64 layers extending from the surface to the mesopause (0.01 hPa ≈ 80 km). The anthropogenic and biomass burning emissions used here were based on the Monitoring Atmospheric Composition and Climate and CityZen (MACCity) emissions dataset and the Global Fire Emissions Database version 3 (GFED3), respectively (Table S2). Concentrations of greenhouse gases were prescribed based on the fifth phase of the Climate Model Intercomparison Project (CMIP5) RCP 6.0 scenario. The reproducibility of surface ozone concentration simulated by MRI-CCM2 was confirmed by comparing with observation data at northern mid-latitude monitoring sites: Waldhof (52.8°N, 10.8°E), Kovk(46.1°N, 15.1°E), Ryori (39.0°N, 141.8°E), Trinidad Head (41.1°N, 235.9°E), Algoma (47.0°N, 275.6°E), and Kejimkujik (44.4°N, 294.8°E) of the WMO World Data Centre for Greenhouse Gases (WDCGG) (http://gaw.kishou.go.jp/wdcgg.html). The six monitoring sites are located in the temperate deciduous forest grids (Fig. S3). Figure S9 shows that MRI-CCM2 can reproduce the seasonal variations in surface ozone at these monitoring sites with a normalized mean error of 0.5–11.9% and a normalized root-mean-square error of 9.0–17.9%.

## Author Contributions

YH analyzed the data for ozone-induced stomatal sluggishness from the experiment performed by YH, MW and TK. MD conducted the global simulations of MRI-CCM2. GK modified SOLVEG to include ozone effects based on parameters obtained by YH and carried out simulations using the result of MD. EP contributed to the analyses. All authors were involved in writing the paper, although YH and EP took a lead role.

## Additional Information

**How to cite this article**: HOSHIKA, Y. *et al*. Ozone-induced stomatal sluggishness changes carbon and water balance of temperate deciduous forests. *Sci. Rep*. **5**, 09871; doi: 10.1038/srep09871 (2015).

## Supplementary Material

Supplementary Information
