# Supplementary material for: Ozone-induced stomatal sluggishness changes carbon and water balance of temperate deciduous forests
Source: Sci Rep. 2015 May 6;5:9871. doi: 10.1038/srep09871 (PMC4421795; doi:10.1038/srep09871)
Supplement: Supplementary Information [file srep09871-s1.doc]

**Supplementary information**

**Ozone-induced stomatal sluggishness changes carbon and water balance of temperate deciduous forests**

Yasutomo HOSHIKA^1,2^, GENKI KATATA^3,4^, Makoto DeUshi^6^, MaKoto WATANABE^5^, TAKAYOSHI KOIKE^1^ and ELENA PAOLETTI^2*^

*1. Silviculture and Forest Ecological Studies, Hokkaido University, Sapporo 060-8689, Japan*

*2. (present address) Institute of Sustainable Plant Protection, National Research Council of Italy, Via Madonna del Piano, I-50019 Sesto Fiorentino, Florence, Italy*

*3. Research Group for Environmental Science, Japan Atomic Energy Agency, 2-4 Shirakata-Shirane, Tokai, Naka, Ibaraki, 319-1195 Japan*

*4. (present address) Atmospheric Environmental Research, Institute of Meteorology and Climate Research, Karlsruhe Institute of Technology, Kreuzeckbahnstr. 19, 82467, Garmisch-Partenkirchen, Germany*

*5. Institute of Agriculture, Tokyo University of Agriculture and Technology, Fuchu 183-8509, Japan*

*6. Atmospheric Environment and Applied Meteorology Research Department, Meteorological Research Institute, Tsukuba, Japan*

*Corresponding author: Elena Paoletti Tel: +39-055-5225-591, Fax: +39-055-5225-666 Email: [elena.paoletti@cnr.it](mailto:elena.paoletti@cnr.it)

**Supplementary Methods**

**A brief description of multi-layer atmosphere-SOil-VEGetation model (SOLVEG)**

SOLVEG is a one-dimensional multi-layer model that consists of four modules for the atmosphere near the surface, and for soil, vegetation, and radiation within the vegetation canopy. The atmosphere module calculates the variables in each atmospheric layer by numerically solving one-dimensional diffusion equations for horizontal wind speed components, potential temperature, specific humidity, liquid water content of the fog, turbulent kinetic energy and length scale, and gas and aerosol concentrations by the second-order turbulence closure model^1^. The soil module calculates soil temperature, volumetric soil water content, and specific humidity of the air in the soil pores using equations for heat conduction, mass balance in liquid water, and water vapor diffusion, respectively^2^. The vegetation module calculates the leaf temperature, the water on the surface of the leaves (leaf surface water) for each canopy layer and the vertical liquid water flux through the entire canopy. In this module, photosynthesis is also incorporated to calculate the CO_2_ assimilation rate based on the relationship between stomatal resistance and the net CO_2_ assimilation rate^3^. The radiation module separately calculates direct and diffuse downward and upward fluxes of solar and long-wave radiation in the canopy and provides the radiation energy input for the heat budget calculations at the soil surface and canopy layers^4^. The basic equations related to gas exchange processes are described in Katata *et al*.^5,6^. Schemes for the deposition of gaseous and particulate matters (including fog droplets) at each canopy layer were incorporated into the model and verified with flux data measured by gradient and eddy covariance methods over semi-arid deserts^2,7^, croplands^4,5,8^, rice paddy field^6^, temperate grasslands^3^, and forests^5,9,10^.

**Ozone exchange process in SOLVEG**

The atmosphere module calculates O_3_ concentration (nmol mol^-1^) by numerically solving the following one-dimensional diffusion equation:

$\frac{\partial c_{\text{O3}}}{\partial t}=\frac{\partial}{\partial z}K_{z}\frac{\partial c_{\text{O3}}}{\partial z}+F_{\text{O3}}$ (S1)

where *t* (s) is time, *z* (m) height of atmosphere, and *K_z_* (m^2^ s^-1^) is vertical turbulence diffusivity calculated by the turbulence closure model^1^. The last term *F*_O3_ is a forcing term, and exchanges between the vegetation and canopy air are considered in this term as the volume source/sink of O_3_. In this study, only the uptake of O_3_ via stomata is considered as *F*_O3_ (Eq. (2)). The model was able to predict the deposition flux of O_3_ due to stomatal uptake over the deciduous broad-leaved forest at Kane experimental site in the USA by considering the uncertainties of plant physiological parameters, non-stomatal deposition, and chemical reaction between O_3_ and soil NO*_x_* emission^5^.

**CO_2_ assimilation and stomatal resistance in SOLVEG**

The net CO_2_ assimilation rate, *A_n_* in Eq. (1), calculated by subtracting the leaf respiration rate *R* (μmol m^-2^ s^-1^) from the assimilation rate, is expressed as^11,12^,

$A_{n}=\min\left(w_{c},w_{e},w_{s}\right)-R$ (S2)

where the CO_2_ assimilation rate is determined as the minimum of three limiting rates (μmol m^-2^ s^-1^), *w_c_* is the limitation in efficiency of Rubisco, *w_e_* is the limitation in the absorbed Photosynthetically Active Radiation (PAR), and *w_s_* is the limitation in the capacity of the leaf to export the products of photosynthesis. These potential rates for C3 plants are calculated as:

$w_{c}=V_{c\max}\frac{c_{i}-\Gamma_{*}}{c_{i}+K_{c}\left(1+O_{2}/K_{o}\right)}$, (S3)

$w_{e}=\alpha f_{PAR}I\frac{c_{i}-\Gamma_{*}}{c_{i}+2\Gamma_{*}}$, and (S4)

$w_{s}=0.5V_{c\max}$, (S5)

where *V_cmax_* is the maximum catalytic capacity of Rubisco (µmol m^-2^ s^-1^), *c_i_* is the CO_2_ partial pressure of leaf interior (Pa), Γ is the CO_2_ compensation point (Pa), *K_c_* and *K_o_* are the Michaelis–Menten constants for CO_2_ and O_2_, respectively, *O_2_* is the O_2_ partial pressure of leaf interior, *a* (µmol-CO_2_µmol-photon^-1^) is the quantum efficiency (=0.06 for C_3_ plants), *f*_PAR_ (µmol-photon J^-1^) is the constant (=4.6) used to convert the PAR unit from (W m^-2^) to (µmol-photon m^-2^ s^-1^), and *I* is the absorbed PAR by unit leaf area (W m^-2^). Note that *V_cmax_* is parameterized as a function of *V_cmax_* at 25 °C (*V_cmax25_*), leaf temperature, and soil moisture^3^. *R* is the respiration rate of leaves (µmol m^-2^ s^-1^), which occurs in both light (*R*_day_) and darkness (*R*_d_). *R*_day_ is assumed to be 0.5*R*_d_^13^. Temperature dependency of *R* can be calculated as^14^,

*R*(*T*_k_)=*R*_25_·exp[{(*T*_k_-298)·d*H*_a_}/(8.314·*T*_k_·298)] (S6)

where *T*_k_ is leaf temperature (K), *R*_25_ is leaf respiration rate at 25 °C, and d*H*_a_ is an energy of activation (45.7 kJ mol^-1^ in Siebold’s beech^13^). A value of 1.1 (µmol m^-2^ s^-1^) for Siebold’s beech was used for dark respiration rate at 25 °C (*R*_d25_) in the present study^15^.

**Supplementary References**

1. Yamada, T. A numerical model study of turbulent airflow in and above a forest canopy. *J. Meteor. Soc. Japan* **60**, 439-454 (1982).

2. Katata, G., Nagai, H., Ueda, H., Agam, N. & Berliner, P.R. Development of a land surface model including evaporation and adsorption processes in the soil for the land–air exchange in arid regions. *J. Hydrometeorol.* **8**, 1307-1324 (2007).

3. Nagai, H. Incorporation of CO_2_ exchange processes into a multilayer atmosphere-soil-vegetation model. *J. Appl. Meteorol*. **44**, 1574-1592 (2005).

4. Nagai, H. Validation and sensitivity analysis of a new atmosphere-soil-vegetation model. Part II: Impacts on in-canopy latent heat flux over a winter wheat field determined by detailed calculation of canopy radiation transmission and stomatal resistance, *J. Appl. Meteorol.* **42**, 434-351 (2003).

5. Katata, G., Nagai, H., Zhang, L., Held, A., Serca, D. & Klemm, O. Development of an atmosphere-soil-vegetation model for investigation of radioactive materials transport in the terrestrial biosphere. *Prog. Nuc. Sci. Tech.* **2**, 530-537 (2011).

6. Katata, G. Hayashi, K., Ono, K., Nagai, H., Miyata, A. & Mano, M. Coupling atmospheric ammonia exchange process over a rice paddy field with a multi-layer atmosphere-soil-vegetation model. *Agr. Forest Meteorol.* **180**, 1-21 (2013).

7. Katata, G., Nagai, H., Kajino, M., Ueda, H., & Hozumi, Y. Numerical study of fog deposition on vegetation for atmosphere–land interactions in semi-arid and arid regions. *Agr. Forest Meteorol.* **150**, 340-353 (2010).

8. Nagai, H. Validation and sensitivity analysis of a new atmosphere-soil-vegetation model. *J. Appl. Meteor.* **41**, 160-176 (2002).

9. Katata, G., Nagai, H., Wrzesinsky, T., Klemm, O., Eugster, W., & Burkard, R. Development of a land surface model including cloud water deposition on vegetation. *J. Appl. Meteor. Climatol.* **47**, 2129-2146 (2008).

10. Katata, G., Kajino, M., Matsuda, K., Takahashi, A. & Nakaya, K. A numerical study of the effects of aerosol hygroscopic properties to dry deposition on a broad-leaved forest. *Atmos. Environ.* in press (2014). 10.1016/j.atmosenv.2013.11.028.

11. Collatz, G.J., Ball, J.T., Grivet, C. & Berry, J.A. Physiological and environmental regulation of stomatal conductance, photosynthesis, and transpiration: a model that includes a laminar boundary layer. *Agric. For. Meteorol.* **54**, 107-136 (1991).

12. Collatz, G.J., Ribas-Carbo, M. & Berry, J.A. Coupled photosynthesis-stomatal conductance model for leaves of C4 plants. *Aust. J. Plant Physiol*. **19**, 519-538 (1992).

13. Iio, A., Fukazawa, H., Nose, Y., Naramoto, M., Mizunaga, H. & Kakubari, Y. Within-branch heterogeneity of the light environment and leaf temperature in a *Fagus crenata* crown and its significance for photosynthesis calculations. *Trees* **23**, 1053-1064 (2009).

14. Bernacchi, C.J., Bagley, J.E., Serbin, S.P. Ruiz-Vera, U.M, Rosenthal, D.M. & Vanloocke, A. Modeling C3 photosynthesis from the chloroplast to the ecosystem. *Plant Cell Environ.* **36**, 1641-1657 (2013).

15. Hoshika, Y., Watanabe, M., Inada, N. & Koike, T. Model-based analysis of avoidance of ozone stress by stomatal closure in Siebold’s beech (*Fagus crenata*). *Ann. Bot.* **112**, 1149-1158 (2013).

16. Yoshida, T. & Kamitani, T. Interspecific competition among three canopy-tree species in a mixed-species even-aged forest of central Japan. *For. Ecol. Manage.* **137**, 221-230 (2000).

17. Kubota, M., Tenhunen, J., Zimmerman, R., Schmidt, M., Adiku, S. & Kakubari, Y. Influences of environmental factors on the radial profile of sap flux density in *Fagus crenata* growing at different elevations in the Naeba Mountains, Japan. *Tree Physiol.* **25**, 545-556 (2005).

18. Kodani, E., Awaya, Y., Tanaka, K. & Matsumura, N. Seasonal patterns of canopy structure, biochemistry and spectral reflectance in a broad-leaved deciduous *Fagus crenata* canopy. *For. Ecol. Manage.* **167**, 233-249 (2002).

19. Masui, T., Matsumoto, K., Hijioka, Y., Kinoshita, T., Nozawa, T., Ishiwatari, S., Kato, E., Shukla, P. R., Yamagata, Y., and Kainuma, M. An emission pathway for stabilization at 6 Wm^−2^ radiative forcing. *Clim. Chan.* **109**, 59–76 (2011).

20. Granier, C. *et al.* Evolution of anthropogenic and biomass burning emissions of air pollutants at global and regional scales during the 1980–2010 period. *Clim. Chan.* **109**, 163–190 (2011).

21. van der Werf, G. R., Randerson, J. T., Giglio, L., Collatz, G. J., Mu, M., Kasibhatla, P. S., Morton, D. C., DeFries, R. S., Jin, Y., & van Leeuwen, T. T. Global fire emissions and the contribution of deforestation, savanna, forest, agricultural, and peat fires (1997–2009). *Atmos. Chem. Phys.* **10**, 11707–11735 (2010).

22. Deushi, M. & Shibata, K. Development of a Meteorological Research Institute Chemistry-Climate Model version 2 for the study of tropospheric and stratospheric chemistry. *Pap. Meteorol. Geophys.* **62**, 1-46, doi:10.2467/mripapers.62.1 (2011).

23. Rayner, N. A., Parker, D. E., Horton, E. B., Folland, C. K., Alexander, L. V., Rowell, D. P., Kent, E. C., & Kaplan, A. Global analyses of sea surface temperature, sea ice, and night marine air temperature since the late nineteenth century. *J. Geophys. Res.* **108(D14)**, 4407 (2003).

24. Schemel, M., Sampson, C., Root, M., & Demeter, M. Temperate deciduous forest: biome summaries, maps, pictures, and climatograms. (2011) https://www.livebinders.com/play/play?id=203716 (Date of access: 02/05/2014).

25. Noormets, A. *et al*. Stomatal and non-stomatal limitation to photosynthesis in two trembling aspen (*Populus tremuloides* Michx.) clones exposed to elevated CO_2_ and/or O_3_. *Plant Cell Environ.* **24**, 327-336 (2001).

**Supplementary table**

**Table S1. Simulation settings for the multi-layer atmosphere–SOil–VEGetation model, SOLVEG.**

| Item | Variable |
| --- | --- |
| Simulation period | 1 May to 1 November for each year from 2006 to 2009 |
| Time step | 6 s |
| Time increment for input meteorological data | 3 hrs |
| Horizontal grid size | 120 km × 120 km |
| Land use | Temperate deciduous broad-leaved forest |
| Soil texture | Loam |
| Numbers of layers | 10, 5, and 7 for atmosphere, vegetation and soil, respectively |
| Soil layer boundaries | 0.02, 0.05, 0.1, 0.2, 0.5, 1.0, and 2.0 m depth |
| Vegetation layer boundaries | 4, 8, 12, 16, and 20 m height |
| Atmospheric layer boundaries | Vegetation layers and 21, 22, 24, 26, 29, and 31 m height |
| Root fraction distribution | Constant to 0.5 m depth |
| Porosity (saturated water content) | 0.43 m^3^ m^-3^ |
| Surface roughness for momentum | 10 mm |
| Surface roughness for heat | 1 mm |
| *V_cmax_* at 25°C | Function of CUO (Eq. 3) |
| *m* | 15 (as an averaged value, see Fig. S1) |
| *g_min_* | Function of CUO (Fig. 1) |
| Canopy height | 20 m (ref. 16, 17) |
| Leaf area index (LAI) | 3 m^2^ m^-2^ (ref. 16-18) |
| CO_2_ concentration | 360 μmol mol^-1^ |
| Other parameters | Same as Katata *et al.*^5^ |

**Table S2. Emissions of trace gases and boundary conditions used for the MRI-CCM2 simulation**

| Item | Emission and boundary data used for the simulation |
| --- | --- |
| Concentrations of greenhouse gases | CMIP5 RCP 6.0 scenario (ref. 19) |
| Anthropogenic emissions | MACCity (ref. 20) |
| Biomass burning emissions | GFED3 (ref. 21) |
| Vegetative, soil and ocean emissions | Same as Deushi and Shibata^22^ |
| Sea surface temperature and sea-ice concentration | HadISST1 (ref. 23) |

**
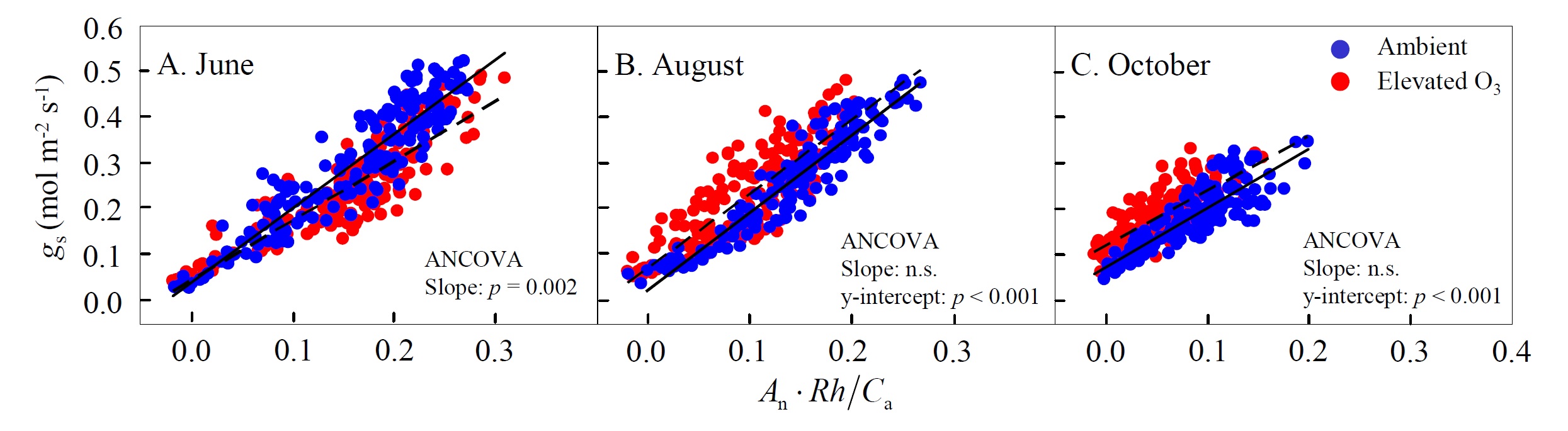
**

**Figure S1. Relationship between stomatal conductance (*g*_s_; mol m^-2^ s^-1^) and the product of net photosynthesis (*A*_n_; μmol mol^–1^) and relative humidity (*Rh*) divided by external CO_2_ concentration (*C*_a_; μmol mol^–1^) of Siebold’s beech in June, August and October under ambient (blue circle and solid line) and elevated O_3_ (red circle and dashed line) at the O_3_ FACE in Japan^15^.** Parameters of Ball-Woodrow-Berry model were calculated as follows: in June, *m* = 16.2 and *g*_min_ = 0.040 mol m^-2^ s^-1^ under ambient O_3_, and *m* = 13.1 and *g*_min_ = 0.044 mol m^-2^ s^-1^ under elevated O_3_; in August, *m* = 16.9 and *g*_min_ = 0.024 mol m^-2^ s^-1^ under ambient O_3_, and *m* = 16.0 and *g*_min_ = 0.074 mol m^-2^ s^-1^ under elevated O_3_; in October, *m* = 12.9 and *g*_min_ = 0.075 mol m^-2^ s^-1^ under ambient O_3_, and *m* = 11.9 and *g*_min_ = 0.121 mol m^-2^ s^-1^ under elevated O_3_.

**
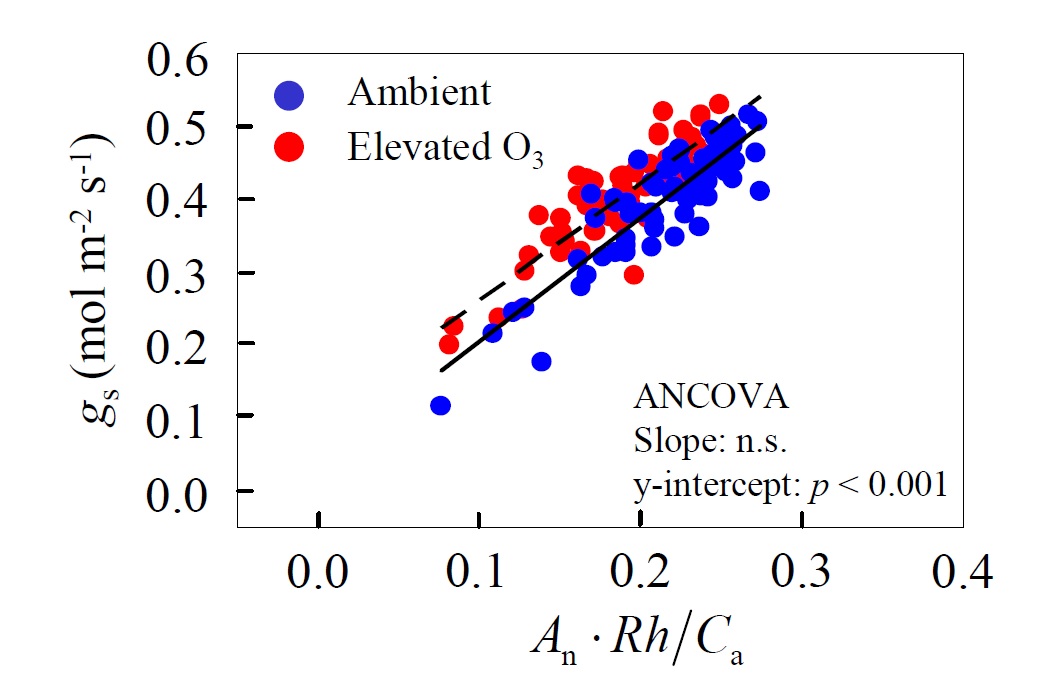
**

**Figure S2. Relationship between stomatal conductance (*g*_s_; mol m^-2^ s^-1^) and the and the product of net photosynthesis (*A*_n_; μmol mol^–1^) and relative humidity (*Rh*) divided by external CO_2_ concentration (*C*_a_; μmol mol^–1^) as obtained from a re-analysis of literature data in Aspen FACE^25^.** O_3_ sensitive Aspen clone leaves were measured in July under ambient O_3_ (blue circle and solid line, *m*=17.1, *g*_min_=0.034 mol m^-2^ s^-1^) and elevated O_3_ (red circle and dashed line, *m*=16.2, *g*_min_=0.100 mol m^-2^ s^-1^).


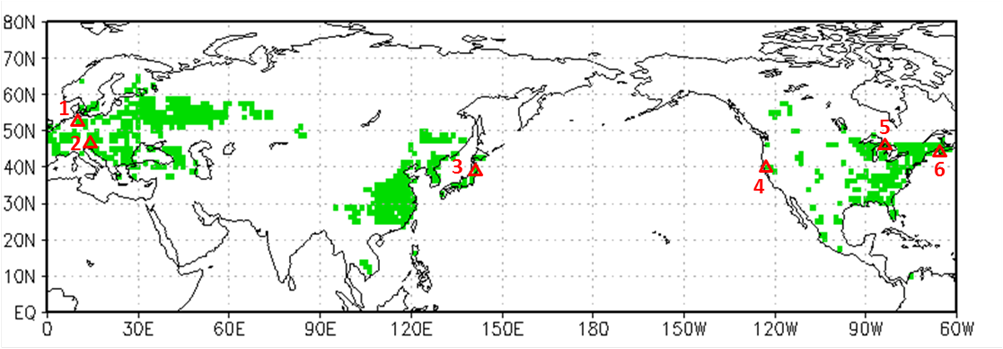


**Figure S3. World map of temperate deciduous forest (green shaded areas) in the Northern Hemisphere for SOLVEG-MRI-CCM2 offline coupling simulation.** The grids were determined from land-use data of JRA55 and reported maps of deciduous forest^24^. Areas surrounded with blue, yellow, black, red, and purple lines represent “Europe”, “Central/West Asia”, “East Asia (without China)”, “China”, and “North America” in Fig. 3, respectively. Only the horizontal grids over the broad-leaved deciduous forest are plotted in the following figures. Red triangles show six WDCGG monitoring sites (1. Waldhof, 2. Kovk, 3. Ryori, 4. Trinidad Head, 5. Algoma, and 6. Kejimkujik), which are located in the temperate deciduous forest grids at northern mid-latitudes. This and following maps are created by Grid Analysis and Display System (GrADS: http://grads.iges.org/grads/index.html).


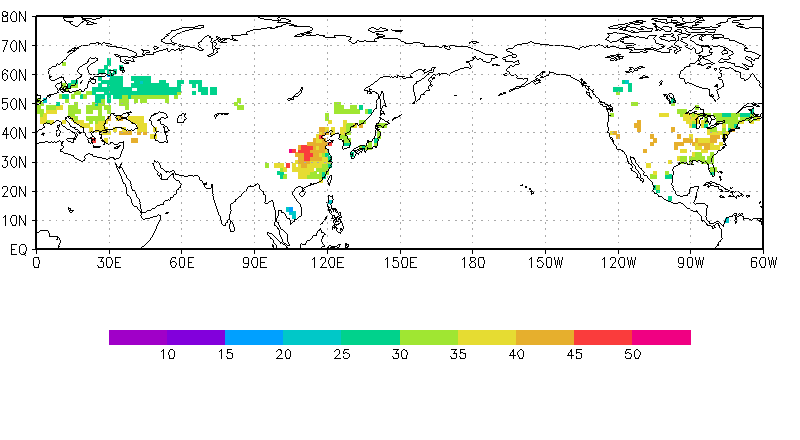

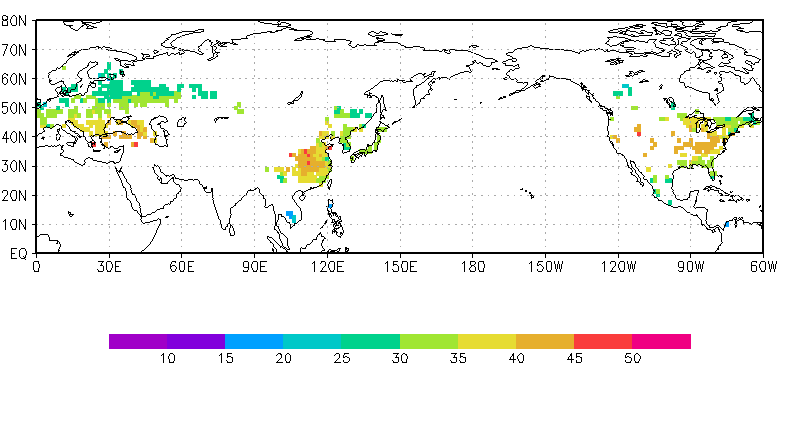

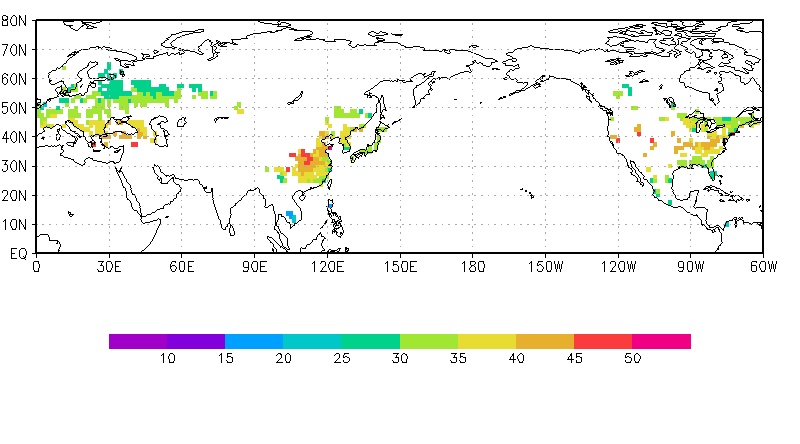

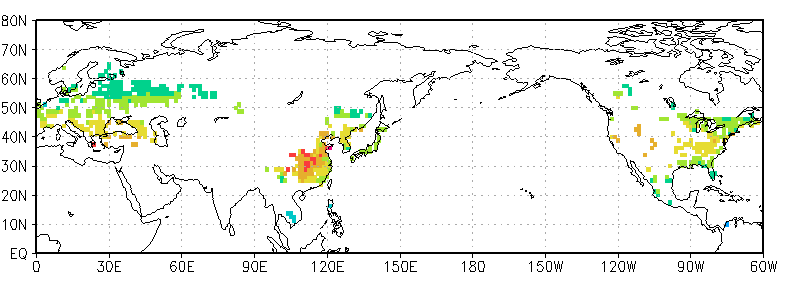

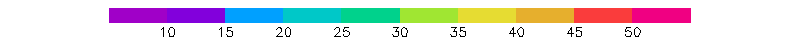


**2006**

**2007**

**2008**

**2009**

(

nmol

mol

-

1

)

**Figure S4. Daily mean ozone concentration in the Northern Hemisphere calculated by MRI-CCM2 for the growing season in 2006–2009.** The growing season is defined as 1 May to 1 November in this study.


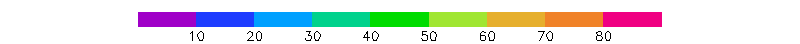

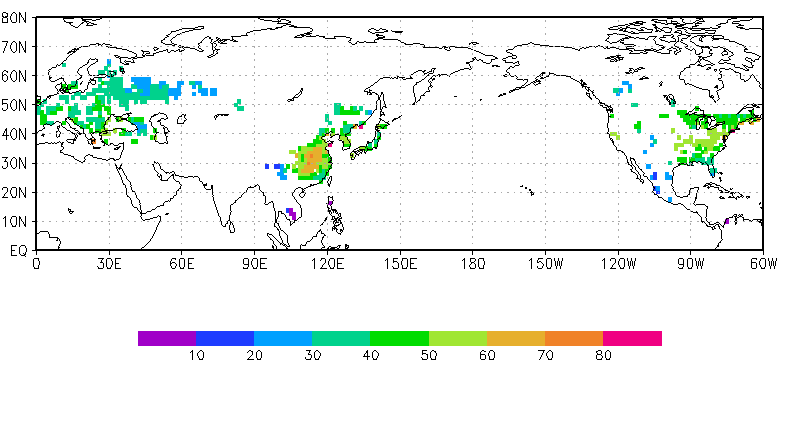

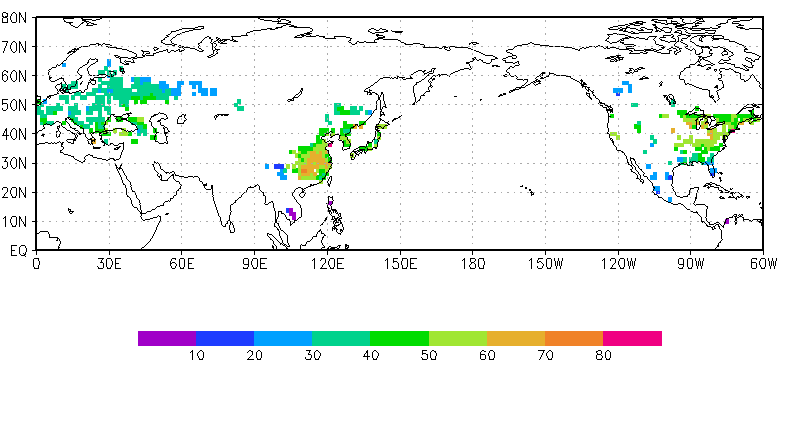

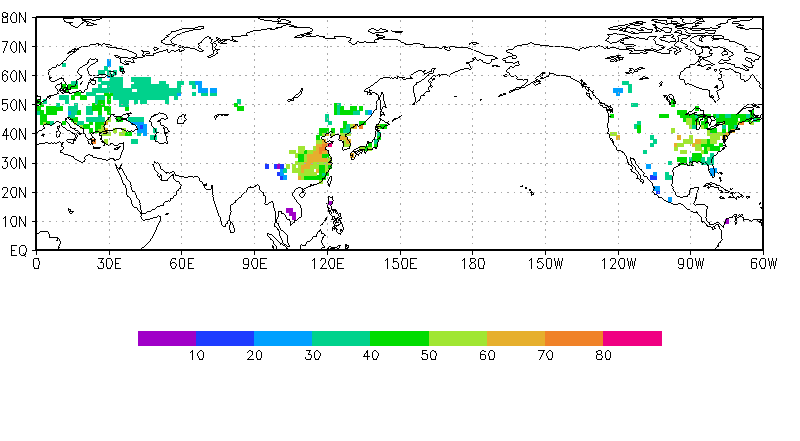

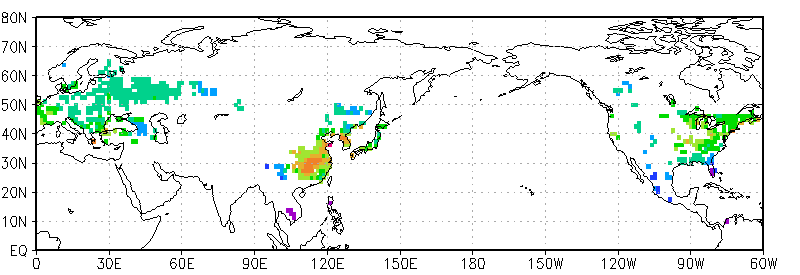


**2006**

**2007**

**2008**

**2009**

(

m

mol

m

-

2

)

**Figure S5. Canopy ozone uptake in the Northern Hemisphere in the offline coupling simulations of SOLVEG-MRI-CCM2 for the growing season in 2006–2009.** The model includes the effect of O_3_-induced stomatal sluggishness (i.e., “sluggishness run”).


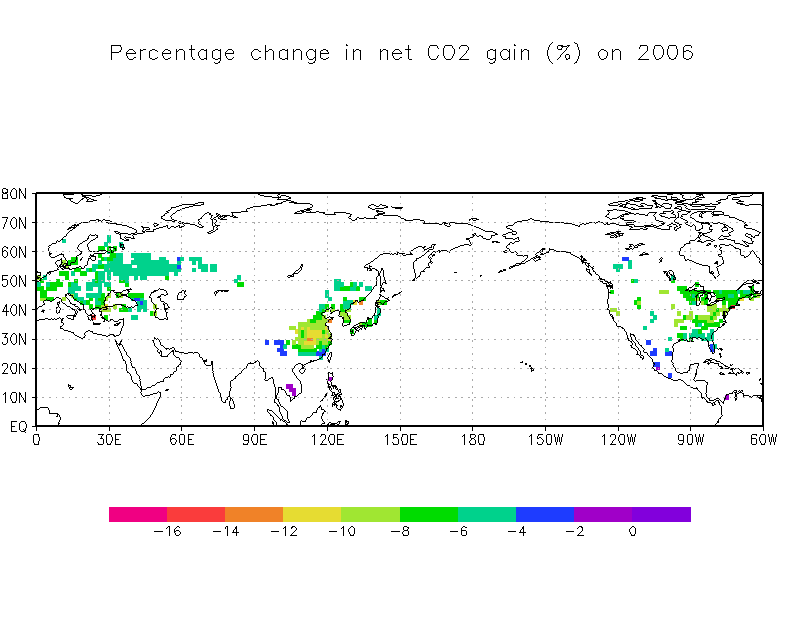

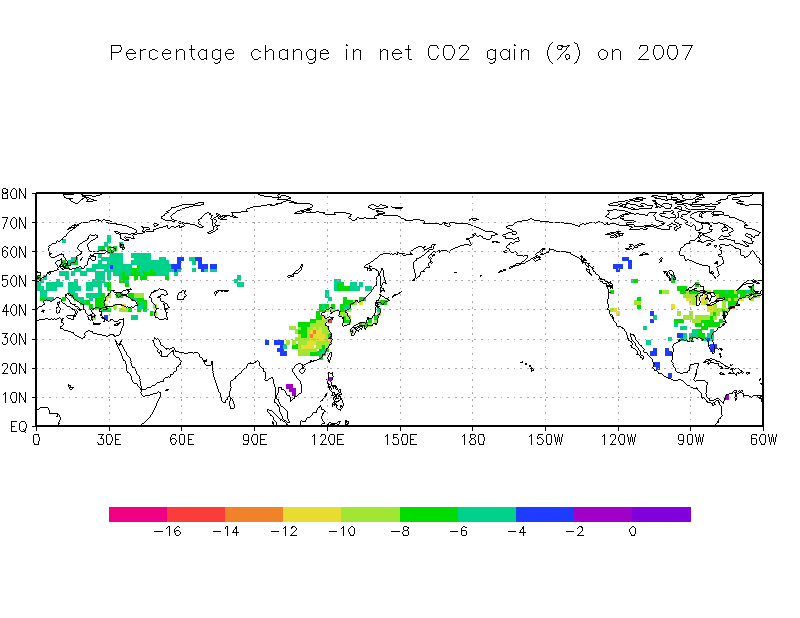

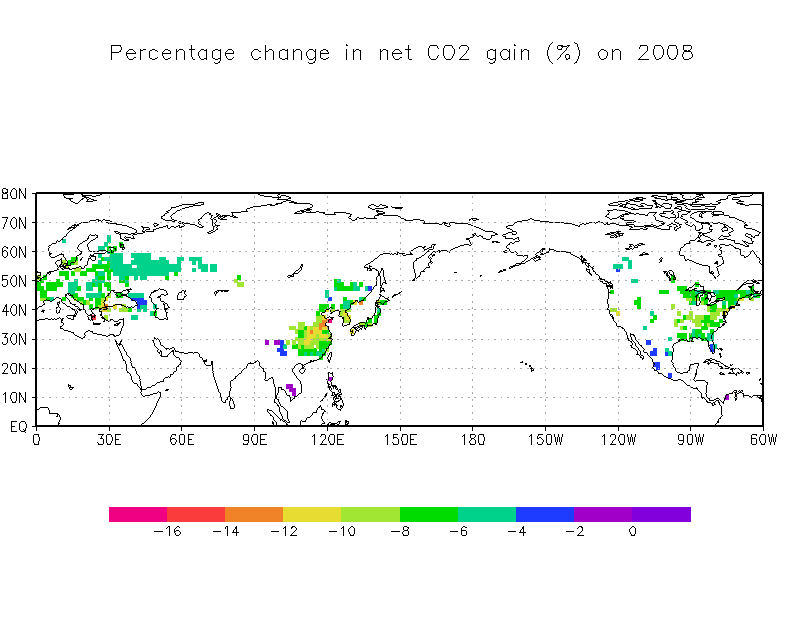

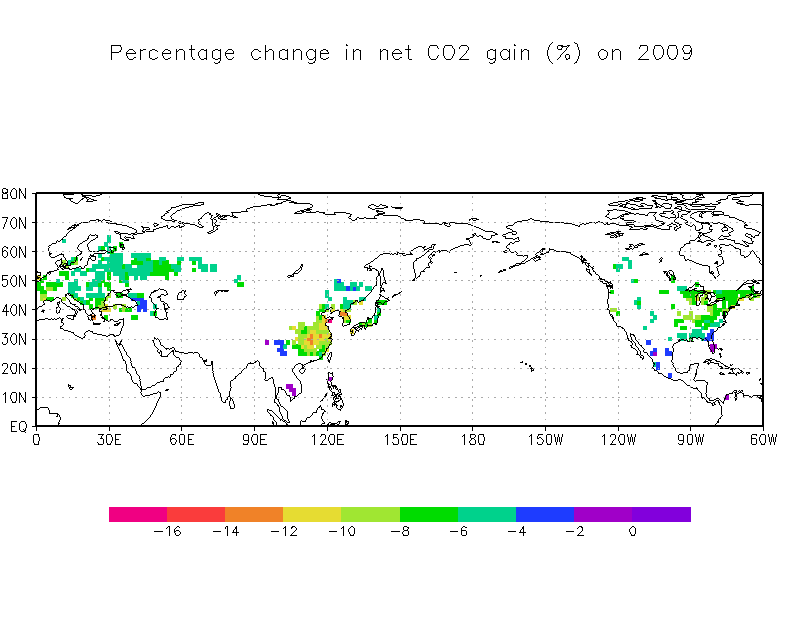

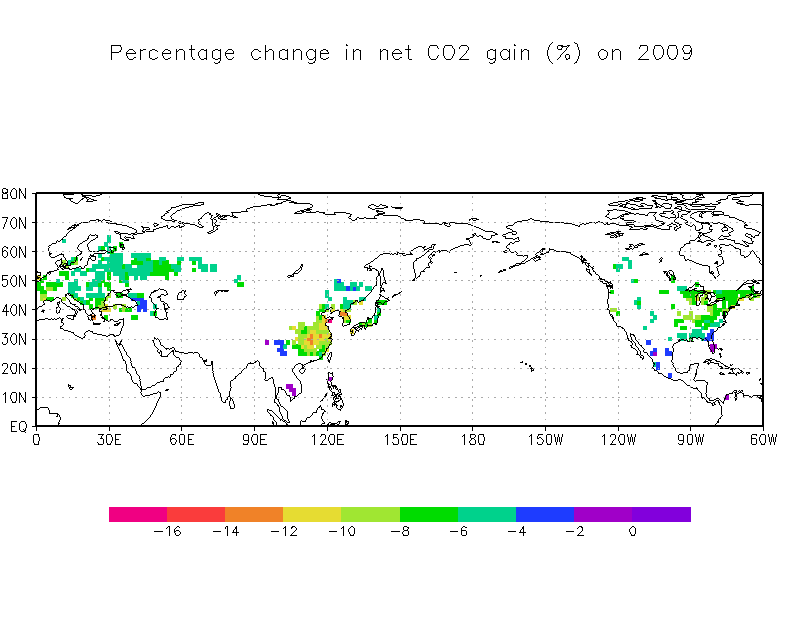


**2006**

**2007**

**2008**

**2009**

(%)

**Figure S6. Percentage change in net CO_2_ assimilation in the Northern Hemisphere calculated in the offline coupling simulations of SOLVEG-MRI-CCM2 for the growing season in 2006–2009.** The model includes the effect of O_3_-induced stomatal sluggishness (i.e., “sluggishness run”). The percentage change was calculated relative to “control run” (no O_3_ effect).


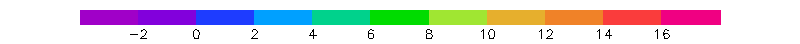

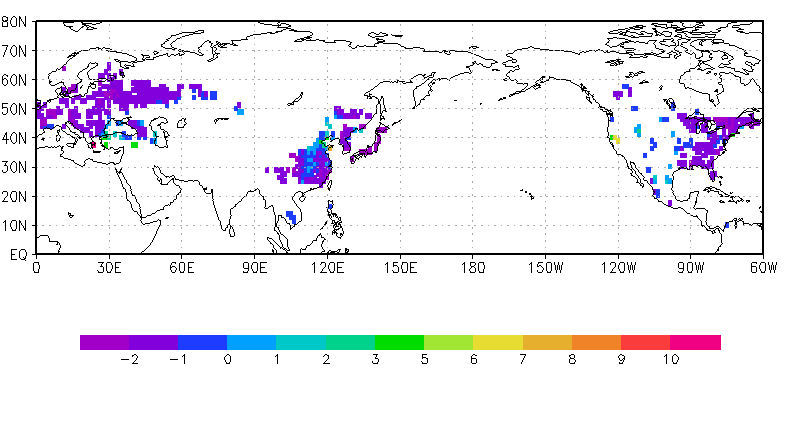

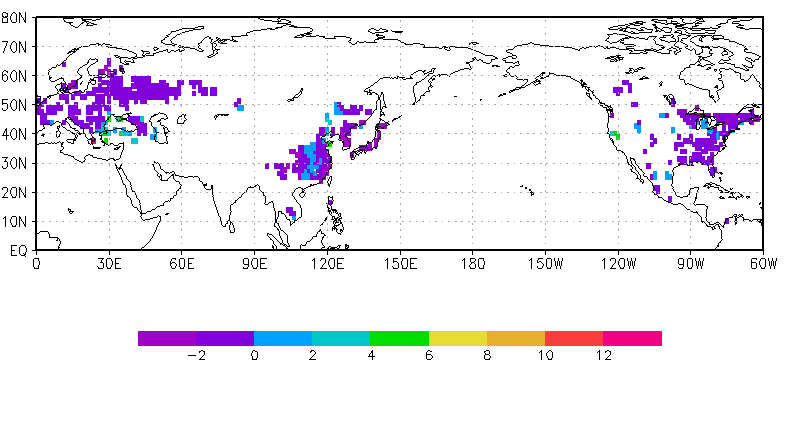

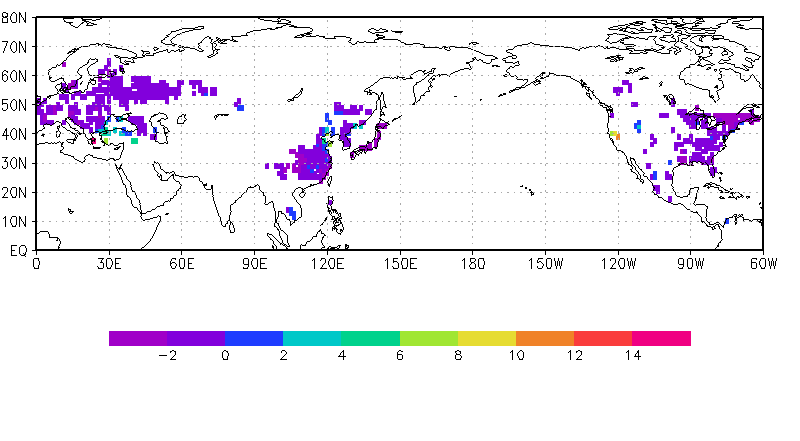

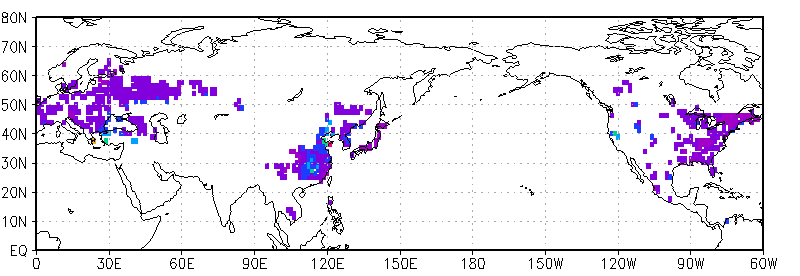


**2006**

**2007**

**2008**

(%)

**2009**

**Figure S7. Percentage change in cumulative transpiration in the Northern Hemisphere calculated in the offline coupling simulations of SOLVEG-MRI-CCM2 for the growing season in 2006–2009.** The model includes the effect of O_3_-induced stomatal sluggishness (i.e., “sluggishness run”). The percentage change was calculated relative to “control run” (no O_3_ effect).


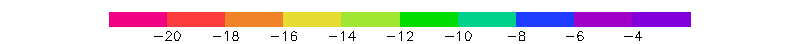

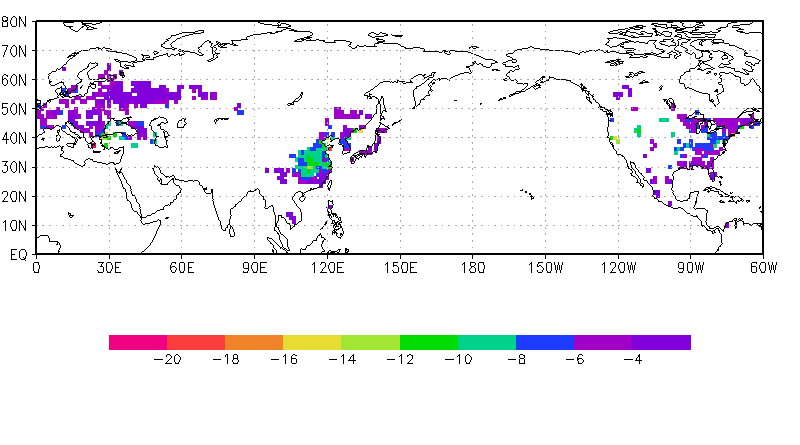

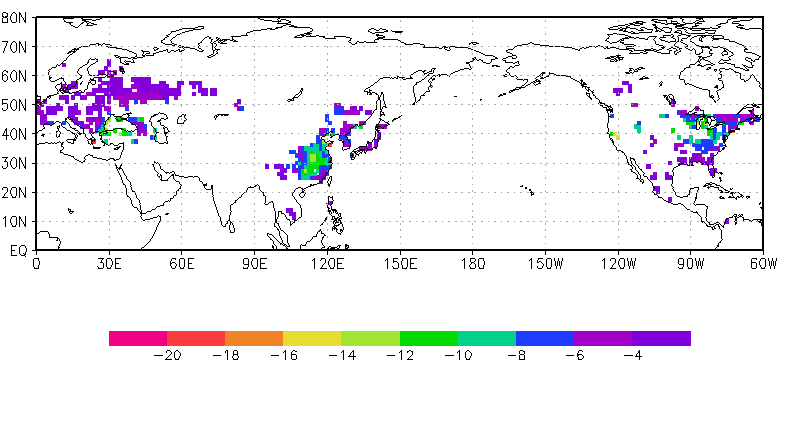

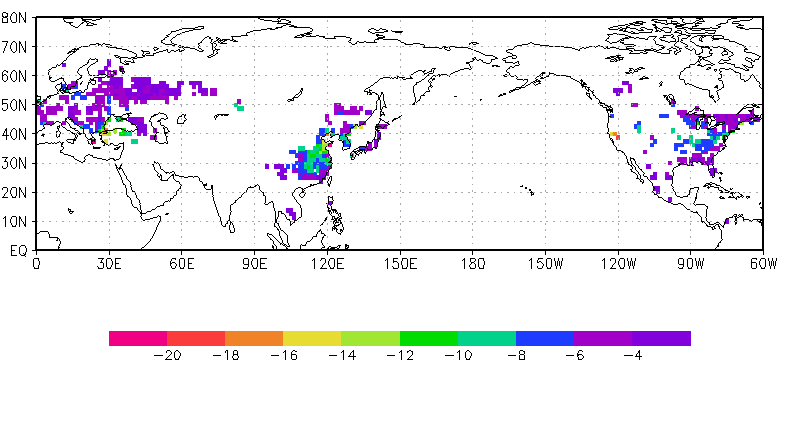

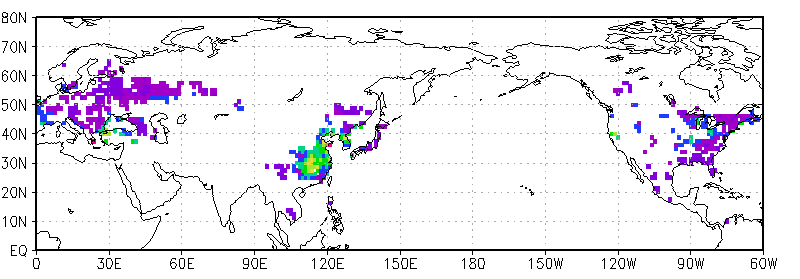


**2006**

**2007**

**2008**

**2009**

(%)

**Figure S8. Percentage change in canopy water use efficiency (WUE) in the Northern Hemisphere calculated in the offline coupling simulations of SOLVEG-MRI-CCM2 for the growing season in 2006–2009.** The model includes the effect of O_3_-induced stomatal sluggishness (i.e., “sluggishness run”). The percentage change was calculated relative to “control run” (no O_3_ effect).


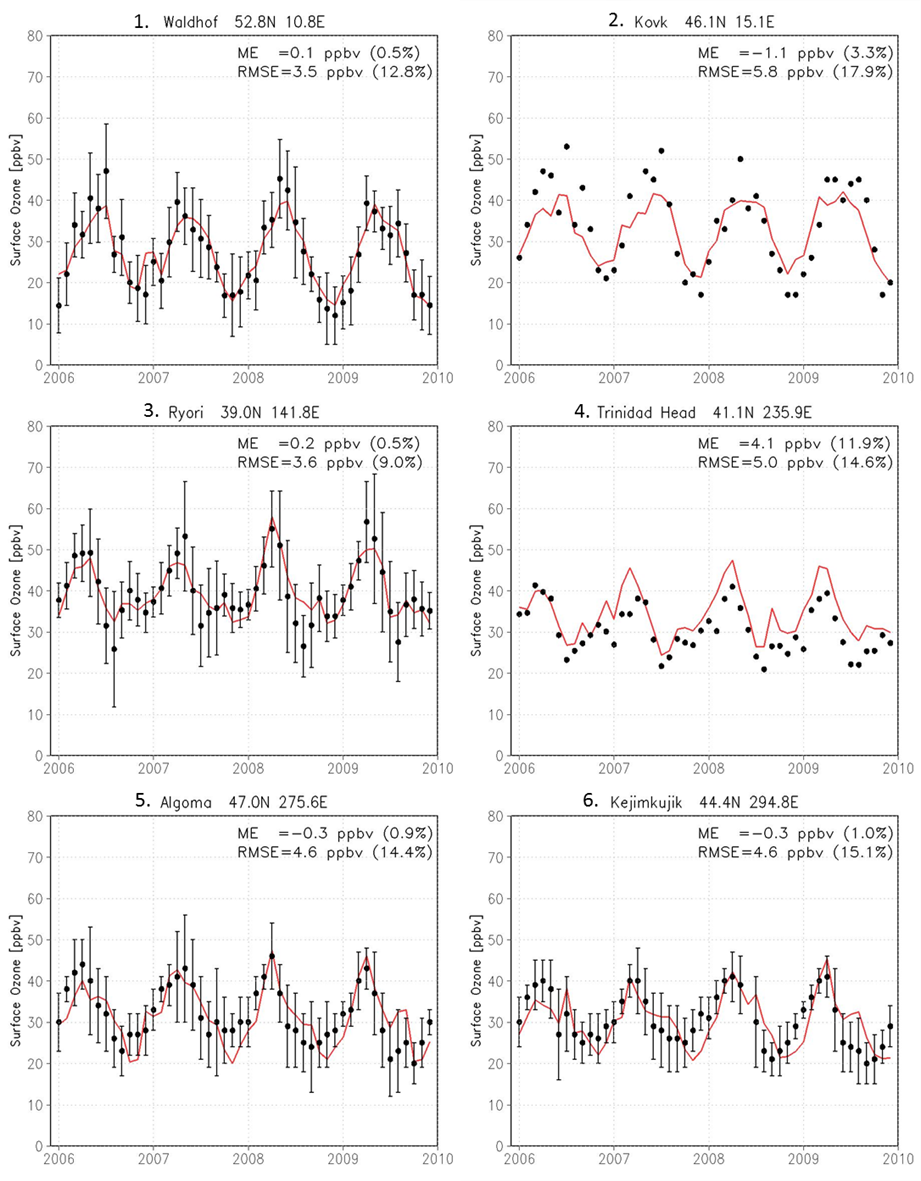


**Figure S9. The monthly averaged observed ozone mixing ratios (in ppbv) (closed circles) with standard deviations (whiskers) at the six WDCGG monitoring sites (1. Waldhof, 2. Kovk, 3. Ryori, 4. Trinidad Head, 5. Algoma, and 6. Kejimkujik** **shown in Fig. S3) in 2006-2009,** **and the simulated mixing ratios (red line) at the corresponding locations.** The mean error (ME) and root-mean-square error (RMSE) of the simulated mixing ratios (in ppbv) are also shown at each monitoring site, with their normalized ones (%) in parentheses.
